# Curcumin Combined with Tryptophan Ameliorates DSS-Induced Ulcerative Colitis via Reducing Inflammation and Oxidative Stress and Regulation of Gut Microbiota

**DOI:** 10.3390/nu17182988

**Published:** 2025-09-18

**Authors:** Hedong Jiang, Gonglong Li, Liuming Xie, Nanhai Zhang, Yi Huang, Xinli Liang, Fanghua Guo, Qieying Jiang, Zhenggen Liao

**Affiliations:** 1 Key Laboratory of Modern Preparation of TCM, Jiangxi University of Chinese Medicine, Nanchang 330004, China; 20201042@jxutcm.edu.cn (H.J.); ligonglong@jxutcm.edu.cn (G.L.); 20131055@jxutcm.edu.cn (X.L.); 2 School of Pharmacy, Jiangxi University of Chinese Medicine, Nanchang 330004, China; xieliuming@jxutcm.edu.cn (L.X.); znh597851323@163.com (N.Z.); 20194031@jxutcm.edu.cn (Y.H.); guofanghua@jxutcm.edu.cn (F.G.); 3 Jiangxi Provincial Key Laboratory of Effective Material Basis of TCM (2024SSY07102), Jiangxi University of Chinese Medicine, Nanchang 330004, China; 4 Experimental Animal Science and Technology Center, Jiangxi University of Chinese Medicine, Nanchang 330004, China; 20050857@jxutcm.edu.cn

**Keywords:** Cur–Trp combination therapy, DSS-induced colitis model, intestinal oxidative balance restoration, gut microbiota, short-chain fatty acid

## Abstract

**Background**: Curcumin (Cur) and tryptophan (Trp) both show promise for treating ulcerative colitis (UC) alone, but their combination has not been explored. This study investigated the therapeutic advantage of the combination (Cur–Trp) for DSS-induced ulcerative colitis in mice. **Methods**: We established a mouse model of ulcerative colitis induced by dextran sulfate sodium (DSS). The mice were treated with Cur, Trp, or Cur–Trp, and to evaluate the therapeutic effects, we assessed clinical signs such as body weight, disease activity index (DAI), and colon length. We also examined intestinal barrier function through indicators including histopathological changes, inflammatory factors, oxidative stress levels, mucin secretion, and tight junction protein expression. Additionally, we analyzed the composition of gut microbiota and the content of its metabolites like short-chain fatty acids (SCFAs). **Results**: The Cur–Trp group produced the most significant improvement, exceeding that of Cur or Trp group. This was evidenced by a significant recovery of this sign, including slower weight loss, reduced colon shortening, and de-creased disease activity index. Compared with the model group, the weight loss of mice in the Cur–Trp group was reduced from 17.15% to 9.73%, which was better than that in the cur group (11.33%) and the Trp group (11.59%). The DAI decreased from the model group (3.6) to the Cur–Trp group (2.4), while the DAI in the Cur group and the Trp group only decreased to 2.9 and 2.8, respectively. The colon length in the Cur–Trp group (6.52 cm) was larger than that in the cur group (6.31 cm), the Trp group (6.23 cm) and the model group (5.5 cm). The Cur–Trp intervention effectively restored intestinal barrier function, as shown by reducing colon tissue dam-age, modulating inflammatory factors, restoring oxidative balance, increasing mucin secretion, and upregulating tight junction protein expression. Further studies showed that the combination uniquely modulated the gut microbiome, increased the Firmicutes/Bacteroidetes (F/B) ratio, decreased the genus of pro-inflammatory bacteria, and in-creased beneficial bacteria, while increasing SCFA levels to alleviate DSS-induced ulcerative colitis. **Conclusions**: Cur–Trp has shown great potential in alleviating colitis and promoting intestinal barrier function, suggesting that the combination of Cur and Trp has the potential to be developed as a therapeutic functional food or dietary supplement for UC. However, more studies are needed to validate this finding. Future research should focus on elucidating the precise molecular mechanisms, optimizing dosage and clinical trials in chronic models and humans to provide more targeted treatment options.

## 1. Introduction

The multifactorial pathogenesis of ulcerative colitis (UC), including genetic susceptibility, epithelial barrier defects, immune response dysregulation and intestinal microbiota dysregulation, generates complex interactions that lead to the long-term persistence of chronic inflammation [1,2]. Epidemiological data indicate that the incidence of UC has increased significantly worldwide, particularly in rapidly industrializing and urbanizing regions, with an annual growth rate of 11.1%, making UC a major global public health challenge [3]. Current clinical treatment mainly relies on corticosteroids, immunosuppressants, and biologics. Although these therapies have shown therapeutic efficacy, long-term use is often accompanied by serious adverse reactions, including increased risk of infection, osteoporosis, and liver and kidney dysfunction [4]. This highlights a pressing clinical demand for novel therapeutic options that are safe, potent, and less invasive. Current strategies often focus on two key areas: first, restoring epithelial barrier integrity, which is typically compromised by diminished mucin-2 (MUC2) secretion and the degradation of tight junction proteins [5,6]. Second, modulating dysregulated innate and adaptive immunity, often indicated by immune cell dysfunction and heightened levels of pro-inflammatory cytokines, which has spurred the use of immunomodulators and targeted biological agents [7,8]. Furthermore, the acknowledgement of the regulatory function of the intestinal microbiota in UC has precipitated research endeavors focused on the development of microbiota-modulating pharmaceuticals and fecal microbiota transplantation [9,10]. However, despite the advancements in monotherapies, clinical response rates remain suboptimal (30~60%), with many patients failing to respond or losing efficacy over time [11]. This limitation highlights the therapeutic limitations of single-target approaches. The pathogenesis of UC is dependent on cross-talk between barrier defects, immune dysregulation, and microbiota dysfunction to initiate and sustain inflammation [12,13].

Given this complexity, combination therapy has become a reasonable strategy for addressing multiple pathological axes. For example, the SONIC trial demonstrated that compared to 44.4% in the monotherapy group, infliximab combined with azathioprine significantly improved the steroid-free clinical remission rate at week 30 to 56.8%, while also reducing the formation of anti-drug antibodies by 60% [14]. Xu et al. [15] used baicalin combined with emodin to treat DSS-induced ulcerative colitis. Both of these compounds were able to inhibit the activity of NF-kB, thereby exerting an anti-inflammatory effect in the experimental system. Notably, their combined use resulted in more significant changes in CD14 and PPAR-γ expression than administration of baicalin or emodin alone, suggesting a potential synergistic effect of the two drugs in modulating these molecular targets associated with inflammatory processes. This successful example demonstrates that combining drugs with different mechanisms of action can overcome the limitations of monotherapy and enhance treatment efficacy [16]. Inspired by this, exploring combination strategies utilizing bioactive compounds derived from natural food sources has emerged as a research hotspot, potentially offering effective therapeutic options while circumventing the adverse effects associated with traditional pharmaceuticals [17].

Curcumin, the main bioactive component of turmeric, is widely consumed in traditional Asian diets and is widely recognized for its powerful anti-inflammatory and antioxidant properties. Extensive in vitro and in vivo studies have shown that curcumin alleviates intestinal inflammation primarily by inhibiting the NF-kB signaling pathway, resulting in reduced expression of pro-inflammatory cytokines such as TNF-α, IL-1β, and IL-6 [18]. In addition, curcumin has been shown to activate PPAR-γ, thereby promoting the secretion of anti-inflammatory factors. In addition, curcumin regulates the gut microbiota by enhancing the abundance of butyrate-producing beneficial bacteria, such as Clostridium and Ruminococcus. Curcumin alleviates intestinal inflammation through a variety of interrelated mechanisms [19]. Clinical studies have demonstrated that curcumin, as an adjuvant to standard UC therapy, significantly improves remission rates and prolongs the duration of remission [20]. Tryptophan is an essential amino acid that is abundant in the daily diet, including in dairy products, eggs, meat, and legumes. Recent research has revealed that tryptophan and its metabolites play crucial roles in maintaining intestinal health. Tryptophan is metabolized by microorganisms to produce indole compounds, which act as ligands for the aryl hydrocarbon receptor (AhR), activating the AhR signaling pathway [21]. The AhR pathway enhances the regenerative capacity of intestinal epithelial cells by inducing IL-22 secretion; upregulates the expression of MUC2 and tight junction proteins (Claudin-1, Occludin), thereby enhancing intestinal barrier function; and promotes the differentiation of regulatory T cells while inhibiting excessive inflammatory responses [22]. Notably, tryptophan metabolism is highly dependent on the function of the gut microbiota, particularly beneficial bacteria such as *Bifidobacterium* and *Lactobacillus* [23,24]. Although both curcumin and tryptophan individually demonstrate protective effects against UC, their synergistic effects when used in combination have not been systematically investigated. Based on existing evidence, The potential for a synergistic relationship between curcumin and tryptophan in alleviating ulcerative colitis warrants investigation. One possible mechanism could involve the modulation of the gut microbiota. Specifically, the established anti-inflammatory and antioxidant effects of curcumin might create a microenvironment conducive to the metabolism of tryptophan by gut bacteria into beneficial compounds like short-chain fatty acids. These metabolites could, in theory, amplify anti-inflammatory pathways and reinforce gut barrier function, suggesting a “component–microbiota–host” network that contributes to the alleviation of colitis. In this network, the curcumin and tryptophan modulate the microbiota, which in turn influences the host’s immune response and gut health, ultimately achieving a synergistic therapeutic effect.

This study explores the novel value of “food component combination therapy” for UC using curcumin combined with tryptophan, based on the core advantages of high safety and easy access of food-derived ingredients, which distinguish them from traditional single-dose or non-food-derived therapies. We will systematically evaluate the therapeutic effect of this combination on DSS-induced UC in mice, focusing on the improvement of clinical symptoms, repair of histopathological damage, restoration of intestinal barrier function, and maintenance of gut microbiota homeostasis. This work aims to provide an alternative, safe, and sustainable food source strategy for UC therapy and to lay the scientific foundation.

## 2. Materials and Methods

### 2.1. Materials and Reagents

Curcumin was provided by Yuanye Bio-Technology Co., Ltd. (Shanghai, China). L-Tryptophan and short-chain fatty acids (SCFAs), including acetate, propionate, butyrate, isobutyrate, valerate, and isovalerate, were obtained from Aladdin Biochemical Technology Co., Ltd. (Shanghai, China). Dextran sulfate sodium (DSS, 36~50 kDa) was acquired from Sigma-Aldrich (St. Louis, MO, USA). Assay kits for superoxide dismutase (SOD), myeloperoxidase (MPO), and malondialdehyde (MDA) were supplied by Nanjing Jiancheng Bioengineering Company (Nanjing, China). Enzyme-linked immunosorbent assay (ELISA) kits for IL-1β, TNF-α, IL-4, IL-6, and IL-10 were from Multisciences (Wuhan, China). Antibodies for MUC-2, ZO-1, and occludin were from Sevier Biotechnology Co., Ltd. (Wuhan, China).

### 2.2. Animal Experimental

**Figure 1:**
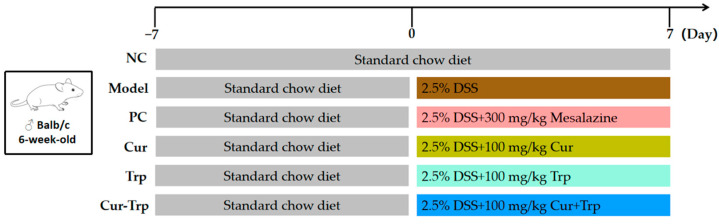
Experimental design and treatment regimen for DSS-induced colitis in mice.

Seventy-two male Balb/c mice (7 weeks old, 22–24 g) were obtained from Jiangsu GemPharmatech Co., Ltd. (Nanjing, China, SCXK 2023-0009). Following a 7-day acclimatization period in a controlled environment (23 ± 2 °C, 12 h light/dark cycle) with ad libitum access to food and water, the mice were randomly allocated into six groups (n = 12) using the RANDBETWEEN function in Excel, including the control group (NC), model group (Model), curcumin group (Cur), tryptophan group (Trp), and Curcumin combined with tryptophan group (Cur–Trp). From day 8, all groups except the NC group received 2.5% dextran sulfate sodium (DSS) in their drinking water to induce colitis. Treatments were administered daily via gavage at a volume of 0.2 mL/10 g body weight. The whole experimental design is illustrated in Figure 1. Throughout the experiment, mice were monitored daily for body weight, fecal characteristics (consistency and blood), and general health status (activity, coat condition, and posture). A humane endpoint was defined as a loss of more than 20% of total body weight, combined with a hunched posture and or reduced activity. Animals or samples were excluded in cases of unexpected death—for instance, due to complications from DSS-induced modeling, errors in gavage administration, or mistakes in sample processing. No unscheduled mortalities occurred during the study period; all deaths resulted from planned euthanasia. Each mouse was handled and treated individually and considered an independent experimental unit for analytical purposes. On day 14, all mice were euthanized by cervical dislocation, and samples of colon tissues, thymus, spleen and cecal contents were collected for subsequent analysis. All procedures were conducted in accordance with the Declaration of Helsinki of the World Medical Association and approved by the Animal Ethics Committee of Jiangxi University of Traditional Chinese Medicine (Approval No. JZLLSC20250520, 20 May 2025).

### 2.3. Disease Activity Index (DAI) Assessment

The Disease Activity Index (DAI) was assessed daily starting from day 8, adapted from the method of Li et al. [25]. The DAI score was calculated as the average of three clinical parameters: weight loss, stool consistency, and rectal bleeding, based on the scoring criteria detailed in Table 1. Rectal bleeding was evaluated by visual inspection and confirmed using a commercial fecal occult blood test kit according to the manufacturer’s instructions.

### 2.4. Intestinal Permeability Analysis

Intestinal permeability was assessed using an in vivo FITC-dextran (4 kDa) following the protocol of Han et al. [26]. Briefly, mice were fasted for 4 h before being administered FITC-dextran (at 400 μg/kg body weight). After 4 h, blood was collected from the tip of the tail vein of the mice, and serum was isolated. The serum samples were then diluted 1:10 (*v*/*v*) with phosphate-buffered saline (PBS). Fluorescence intensity was measured using a microplate reader at an excitation wavelength of 485 nm and an emission wavelength of 535 nm. A standard curve, generated from serial dilutions of FITC-dextran in PBS, was used to quantify the FITC-dextran concentration in the serum.

### 2.5. Histological and Immunofluorescence Analyses

The histological analysis was determined according to the method of Xu et al. [27]. Colonic tissue samples were fixed in 4% formaldehyde, embedded in paraffin, and cut into 5 µm sections. The sections were stained using the hematoxylin and eosin (H&E) method. Stained slides were then examined under an inverted microscope to assess and score histopathological damage based on the criteria outlined (Table 2). For immunofluorescence analysis, paraffin-embedded colon sections were deparaffinized and rehydrated. Antigen retrieval was performed by heating the sections in a citrate buffer. To block non-specific binding, the sections were incubated with 10% BSA for 30 minutes at room temperature. Subsequently, the sections were incubated overnight at 4 °C with primary antibodies against ZO-1, Occludin, and MUC-2. After washing, the sections were incubated with a corresponding fluorescently-labeled secondary antibody for 1 hour at room temperature in the dark. Finally, the stained sections were mounted and examined under a fluorescence microscope. The fluorescence intensity of ZO-1, Occludin, and MUC-2 was quantified using ImageJ software 1.53v (National Institutes of Health, Bethesda, MD, USA).

### 2.6. Oxidative Stress and Inflammatory Cytokine

Colon tissue homogenates were used to assess both oxidative stress markers and inflammatory cytokines. For oxidative stress, the activities of myeloperoxidase (MPO), superoxide dismutase (SOD), and the levels of malondialdehyde (MDA) were measured using corresponding commercial assay kits (Nanjing Jiancheng Bioengineering Institute, Nanjing, China). For inflammation, we quantified the protein concentrations of tumor necrosis factor-alpha (TNF-α), interleukin-6 (IL-6), interleukin-1β (IL-1β), interleukin-4 (IL-4), and interleukin-10 (IL-10). This was performed with enzyme-linked immunosorbent assay (ELISA) kits (MultiSciences (Lianke) Biotech Co., Ltd, Hangzhou, China), strictly adhering to the provided protocols.

### 2.7. SCFAs Analysis

Cecal contents (100 mg) were precisely weighed and homogenized in 1 mL of sterile saline containing stainless steel beads using a tissue lyser (70 Hz, 180 s). The homogenate underwent centrifugation (10,000× *g*, 10 min, 4 °C) to obtain supernatant. Subsequently, 0.8 mL of supernatant was acidified with 0.2 mL sulfuric acid (50% v/v) through vortex mixing (45 s), followed by extraction with 0.95 mL anhydrous ethyl ether for short-chain fatty acid (SCFA) isolation. The organic phase was filtered through 0.22 μm organic membrane filters prior to gas chromatography (GC) analysis (Agilent 6890N; Agilent Technologies, Santa Clara, CA, USA). Chromatographic separation was performed using a DB-FFAP capillary column (30 m×0.25 mm× 0.25 µm, Agilent) with an injection volume of 1 µL and a split ratio of 10:1. The temperature gradient protocol was programmed as follows: initial temperature held at 80 °C for 0.5 min, followed by a ramp of 30 °C/min to 140 °C (0.5 min hold), then a 5 °C/min ramp to 190 °C (0.5 min hold), and finally a rapid ramp of 60 °C/min to 230 °C (1 min hold). A multi-point calibration curve was constructed using serial dilutions of SCFA standards (acetate, propionate, butyrate), and fecal SCFA concentrations were quantified by correlating sample peak areas with the calibration curve (linear regression, R**^2^** > 0.995).

### 2.8. 16SrRNA Sequencing

Cecal content samples were submitted to Novogene Co., Ltd. (Beijing, China) for 16S rRNA gene sequencing. This includes DNA extraction, PCR amplification, magnetic bead purification and recovery, fluorescence quantification, library preparation and high-throughput sequencing. The total genomic DNA of the bacteria was extracted using the E.Z.N.A. **^®^** fecal DNA kit (D4015; Omega Bio-tek, Inc., Norcross, GA, USA), and used as a template for the hypervariable region V3–V4 of the bacterial 16S rRNA gene with 341F(5′-CCTAYGGGRBGCASCAG-3′) and 806R(5′-GGACTACHVGGGTWTCTAAT-3′) primer pairs. The PCR products were purified by AM Pure XT beads (Beckman Coulter Genology, Danvers, MA, USA), and then the PCR products were quantified by Qubit (Invitrogen; Thermo Fisher Scientific, Waltham, MA, USA). After purification, the sequencing data, including principal coordinate analysis (PCoA), α-diversity analysis, non-metric multidimensional scaling (NMDS), and differential bacterial abundance analysis were performed via the Novogene company’s cloud platform (www.Novogene.com, accessed on 25 July 2024).

### 2.9. Statistical Analysis

Data are presented as the mean ± standard deviation (SD). Statistical analyses were performed using SPSS software (version 27.0, IBM, Armonk, NY, USA). Group differences were assessed by one-way analysis of variance (ANOVA) followed by Dunnett’s post-hoc test. A *p*-value less than 0.05 was considered statistically significant. All figures were generated using GraphPad Prism (version 9.5.0, GraphPad Software, San Diego, CA, USA).

## 3. Results

### 3.1. Curcumin Combined with Tryptophan Mitigated the Symptoms of UC

**Figure 2:**
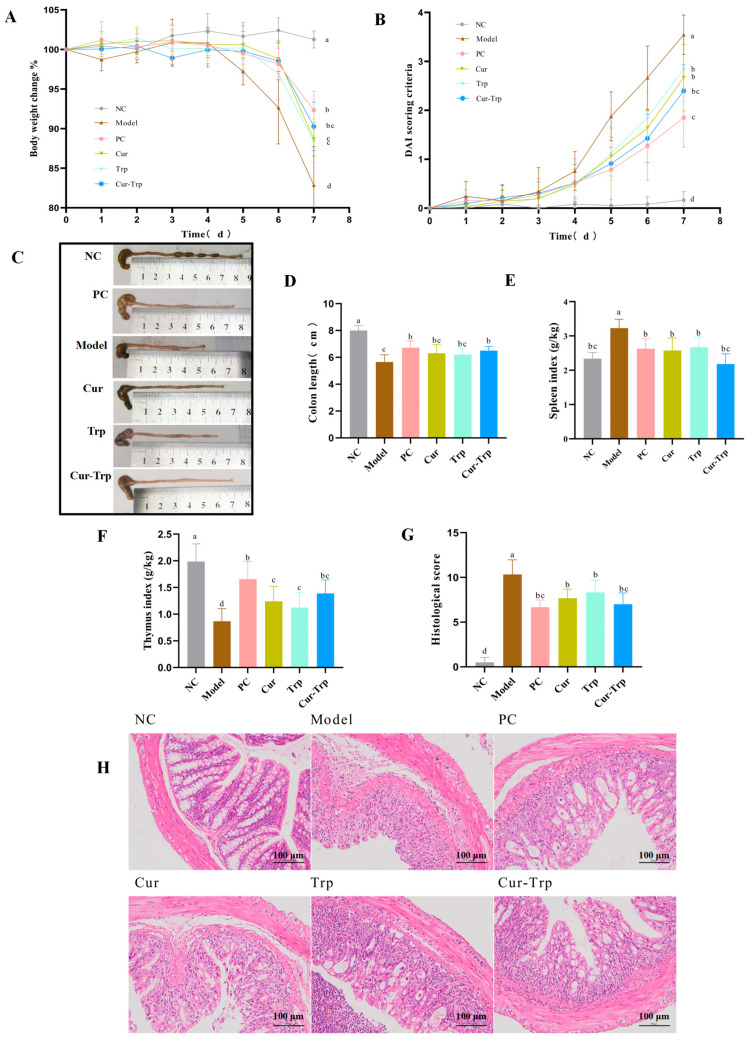
Curcumin combined with tryptophan alleviated symptoms in DSS-induced UC mice, alleviating colon injury. (**A**) Daily weight change; (**B**) DAI score results; (**C**) photos of mouse colons in different groups; (**D**) results of colon length; (**E**) spleen index; (**F**) thymus index; (**G**) histopathological score of colonic tissues; (**H**) representative photographs of H&E staining of colonic tissues (magnification ×200). Values not sharing a common superscript letter denote significant difference (*p* < 0.05).

Continuous administration of 2.5% DSS successfully induced colitis in mice, characterized by clinical signs such as diarrhea, hematochezia, and significant weight loss (Figure 2A). Mice in all DSS-treated groups experienced a progressive decline in body weight. Specifically, body weight decreased in all experimental groups. The most significant weight loss was observed in the Model group (17.15%), followed by the Trp (11.59%), Cur (11.34%), Cur–Trp (9.73%), and PC (7.67%) groups. Compared to the Model group, all treatment groups exhibited significantly attenuated body weight loss. Notably, the Cur–Trp group demonstrated a more pronounced effect in preventing weight loss than the Cur or Trp groups. Meanwhile, the DAI scores of PC (1.85 ± 0.60), Cur–Trp (2.49 ± 0.48), Cur (2.67 ± 0.68), and Trp (2.85 ± 0.67) mice were significantly reduced compared to Model group (3.55 ± 0.40) (Figure 2B). Meanwhile, the immune organ index of the Cur–Trp group showed divergent changes (Figure 2E,F). Specifically, the spleen index of the Cur–Trp group was lower than that of either the Cur or Trp group. In contrast, its thymus index was higher than that of the single-treatment groups. These results indicate that Cur–Trp ameliorates colitis in mice and is superior to monotherapy.

### 3.2. Curcumin Combined with Tryptophan Relieved Colonic Injury

A histology test was used to observe the histopathological status of the colon (Figure 2C,D). Persistent inflammation and ulcer irritation in UC can lead to narrowing and shortening of intestinal passages, and the length of colon can reflect the severity of UC to some extent. In the NC group, the colon tissue exhibited normal color without hyperemia, normal thickness without edema, and no adhesion to surrounding tissues. The colons of Model group mice showed congestion, swelling, and significant shortening. The colon lengths of mice in the Cur (6.31 ± 0.54 cm), Trp (6.20 ± 0.49 cm), Cur–Trp (6.42 ± 0.36 cm), and PC groups (6.72 ± 0.41 cm) were longer than that in the Model group (5.66 ± 0.52 cm), though shorter than that in the NC group, indicating that all interventions alleviated colon shortening, albeit with varying efficacies (Figure 2D). The Cur–Trp group exhibited a more pronounced therapeutic effect. Under the action of H&E staining (Figure 2G,H), it was evident that the epithelial cell surface of the NC group was intact, and the tissue structure was relatively complete. The intestinal mucosa layer and crypts of the NC mouse were also clearly discernible. The intestinal tract architecture in the normal state was compromised following DSS consumption, with the inflamed area infiltrated by an increased number of inflammatory cells. In comparison with the Model group, the Cur, Trp, and Cur–Trp mice exhibited relative tissue integrity and a reduced degree of inflammation. Furthermore, the number of epithelial cells that detached was reduced in the Cur–Trp group, and the Cur–Trp group exhibited a slight invasion of inflammatory cells. The present study demonstrates that the combination of curcumin and tryptophan is more efficacious in restoring and protecting colon lengths than either agent alone. Furthermore, this combination alleviates the damage induced by DSS in murine intestines.

### 3.3. Curcumin Combined with Tryptophan Protected Oxidative Stress

**Figure 3:**
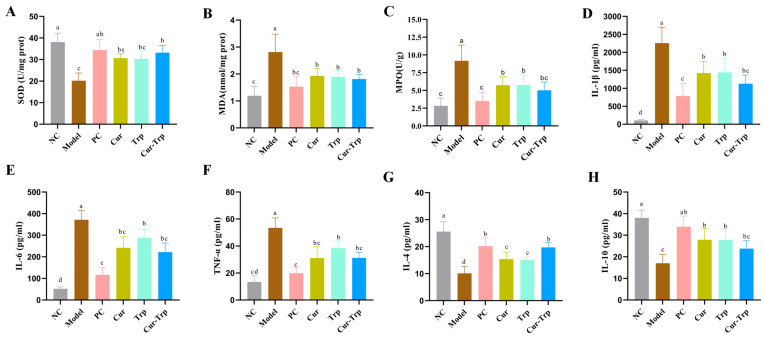
Curcumin combined with tryptophan ameliorates colonic oxidative stress and inflammation in DSS-induced colitis. (**A**) SOD; (**B**) MDA; (**C**) MPO; (**D**) IL-1β; (**E**) IL-6; (**F**) TNF-α; (**G**) IL-4; (**H**) IL-10. Values not sharing a common superscript letter denote significant difference (*p* < 0.05).

To evaluate the effect of curcumin and tryptophan on oxidative stress in DSS-induced colitis, we measured the levels of MDA, MPO and SOD in the colon (Figure 3A–C). The DSS Model group exhibited a significant increase in MDA and MPO levels and a simultaneous decrease in SOD activity compared to the NC group (*p* < 0.05), indicating a severe state of oxidative imbalance. Conversely, Cur, Trp, and Cur–Trp treatments all mitigated these changes. Specifically, these interventions significantly reduced elevated MDA and MPO levels, while restoring SOD activity. Notably, the Cur–Trp group showed the most significant effect in rebuilding colonic oxidative homeostasis, showing superior therapeutic benefit compared to treatment alone.

### 3.4. Curcumin Combined with Tryptophan Improved the Inflammatory Status

The content of pro-inflammatory factors (TNF-α, IL-1β and IL-6) increased significantly and two anti-inflammatory cytokines (IL-10 and IL-4) were significantly reduced under DSS induction (Figure 3D,H); however, the Cur, Trp and Cur–Trp treated groups all alleviated these conditions. Compared with the Cur group and Trp group, the combined administration group showed a more remarkable effect *(p* < 0.05). This suggests that the Cur–Trp group exhibited better anti-inflammatory activity in modulating inflammatory factors in the colon. In summary, Cur–Trp has the ability to reduce colon inflammation in mice by modulating the expression of inflammatory factors.

### 3.5. Curcumin Combined with Tryptophan Improved the Intestinal Barrier Function

**Figure 4:**
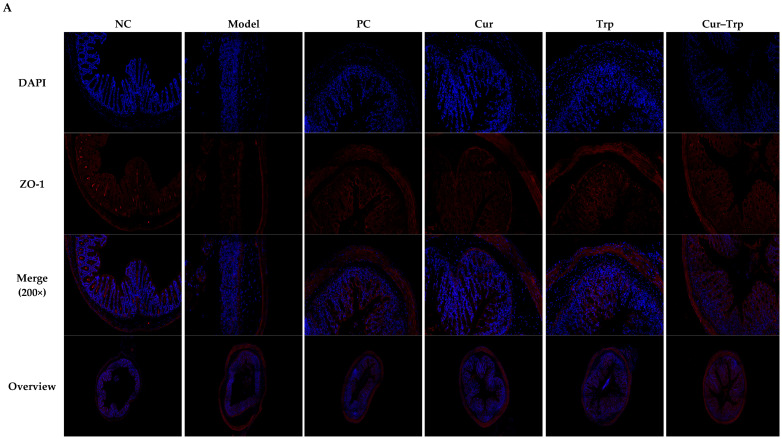

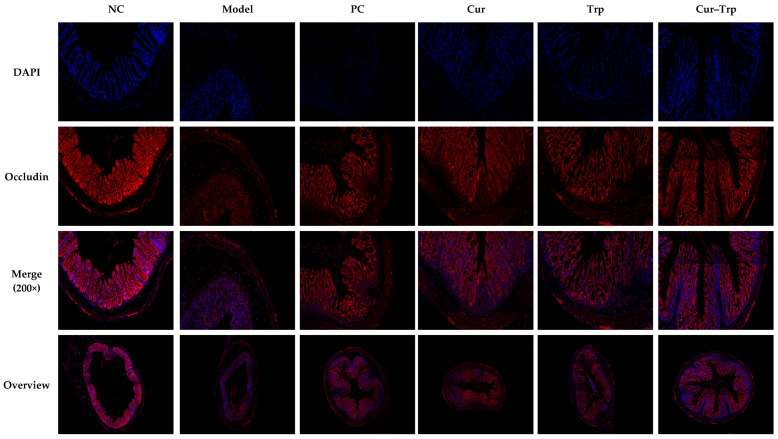

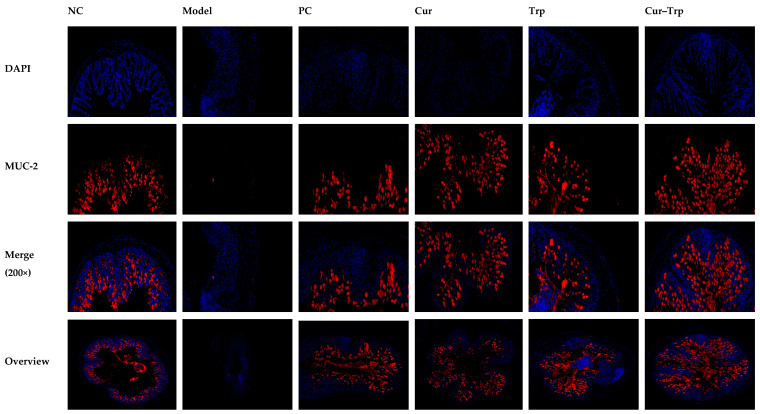

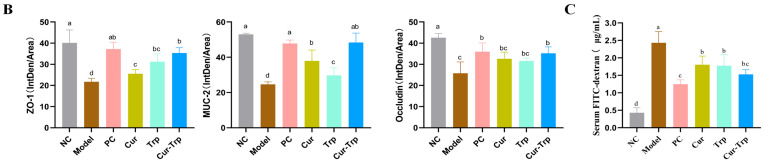
Curcumin combined with tryptophan restores intestinal barrier integrity in DSS-induced colitis. (**A**) Fluorescence image of ZO-1, occludin and MUC-2. Target proteins are shown in red. Scale bars = 100 μm. Magnification, 200×; (**B**) fluorescence quantitative results of ZO-1, occludin and MUC-2; (**C**) intestinal permeability as measured by serum concentrations of FITC-dextran. Values not sharing a common superscript letter denote significant difference (*p <* 0.05).

To evaluate the protective effects of curcumin and tryptophan on the intestinal barrier, we examined the expression of tight junction protein (ZO-1, occludin) and mucin (MUC-2), and measured intestinal permeability. Immunofluorescence results showed that the expression of ZO-1, occludin, and MUC-2 was significantly reduced in the DSS-induced model group compared to the NC group. In contrast, the intervention of Cur, Trp, and especially Cur–Trp effectively reversed this downregulation and restored the expression of these key barrier-related molecules (Figure 4A,B). These protein expression changes were supported by data on intestinal permeability. The FITC-dextran assay showed a significant increase in serum FITC-dextran concentrations in the model group, confirming impaired intestinal barrier function (Figure 4C). This barrier disruption was mitigated in all treatment groups, with serum FITC-dextran levels significantly lower than in the model group. Importantly, Cur–Trp group showed the most prominent decrease in permeability, reflecting the strongest ability to repair the intestinal barrier (*p* < 0.05). In conclusion, these results suggest that curcumin and tryptophan exert a synergistic effect by upregulating the expression of key structural proteins, enhancing gut barrier integrity.

### 3.6. Curcumin Combined with Tryptophan Increased SCFA Content

**Figure 5:**
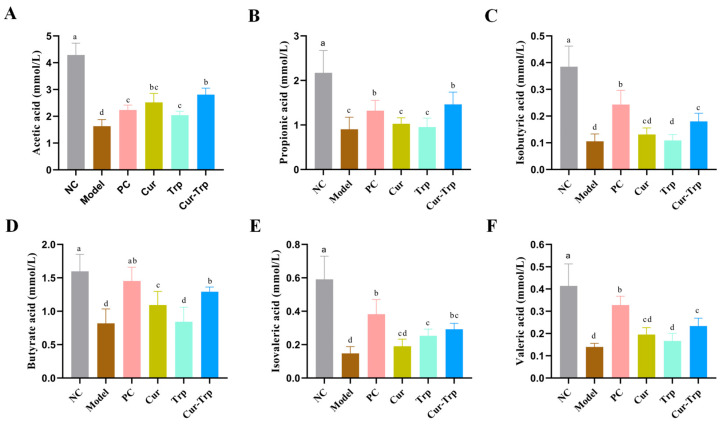
SCFAs content in cecal samples. (**A**) Acetic acid; (**B**) propionic acid; (**C**) isobutyric acid; (**D**) butyrate acid (**E**) isovaleric acid; (**F**) valeric acid. Values not sharing a common superscript letter denote significant difference (*p* < 0.05).

Administration of DSS resulted in significant depletion of all six SCFAs compared to the normal control (NC) group. In contrast, supplementation with Cur, Trp, and Cur–Trp restored SCFA levels. Although both curcumin and tryptophan monotherapy partially restored SCFA levels, the synergistic Cur–Trp group showed the strongest effect, significantly increasing all measured SCFA concentrations to levels much higher than those seen in the Cur or Trp groups alone (*p* < 0.05) (Figure 5). These results suggest that supplementation with Cur–Trp has been shown to be an effective strategy for improving the state of DSS-induced SCFA reduction.

### 3.7. Curcumin Combined with Tryptophan Altered Gut Microbiota

**Figure 6:**
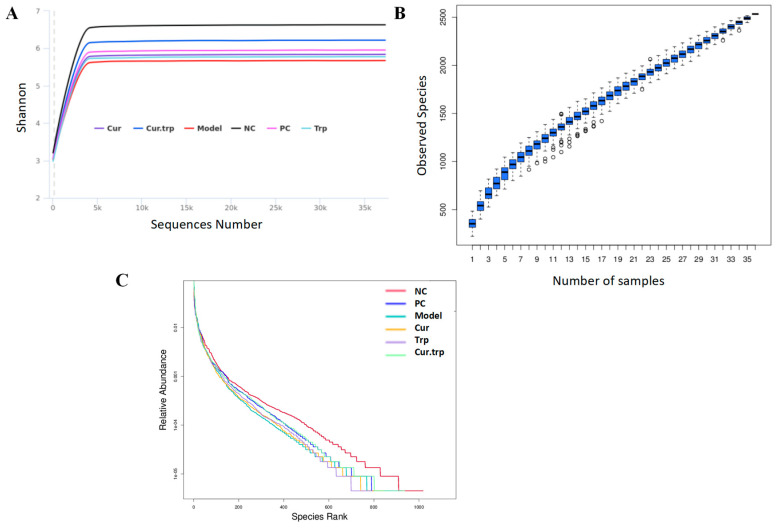
(**A**) Rarefaction cure; (**B**) Species accumulation Curve; (**C**) Rank abundance curve.

The quality and adequacy of the sequencing data were evaluated to ensure robust downstream analysis (Figure 6). The rarefaction curves (Figure 6A), which plot the number of observed species against sequencing depth, all approached a plateau. This saturation indicates that the sequencing depth was sufficient to capture the majority of the microbial diversity within each sample. The species accumulation curve (Figure 6B), showing the number of unique species as a function of the number of samples, also began to level off. This suggests that the overall sample size was adequate to represent the microbial diversity across the experimental groups. The rank abundance curves were plotted to visualize the richness and evenness of the microbial communities (Figure 6C). The horizontal span of the curves reflects species richness, while the slope of the curves indicates species evenness. Collectively, the OTU data from each sample adequately represent the abundance and evenness of gut species.

**Figure 7:**
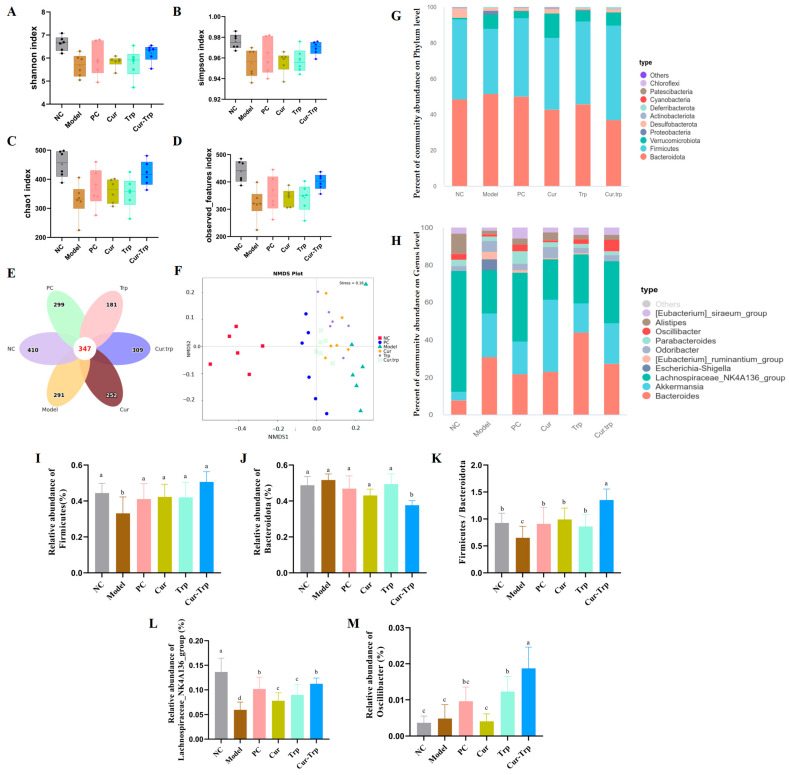

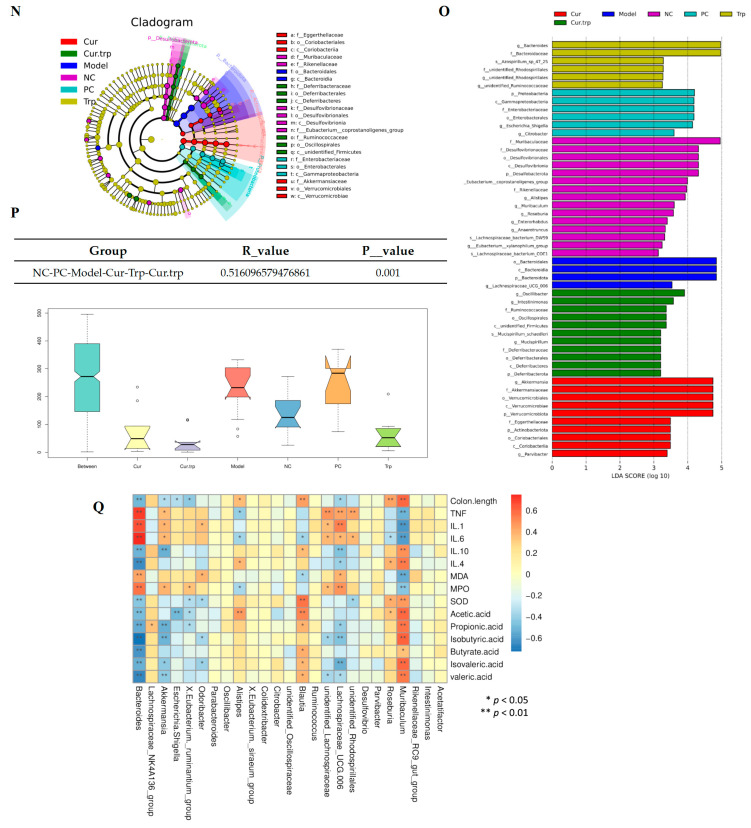
Curcumin combined with tryptophan altered gut microbiota. (**A**–**D**) The Shannon, Simpson, Chao1, and Observed indices; (**E**) Venn diagram of common and unique OTUs among different groups; (**F**) NMDS analysis of intestinal microbiota in different groups; (**G**,**H**) the taxonomic composition at the phylum and genus levels for each group, respectively; (**I**,**J**,**L**,**M**) different groups of differential microbial taxa at the phylum or family level; (**K**) the ratio of Firmicutes/Bacteroidetes (F/B) at the phylum level; (**N**) taxonomic cladogram generated from default LEfSe analysis; (**O**) LDA histograms, LDA score > 3; (**P**) ANOSIM analysis; (**Q**) Spearman’s correlation analysis. Values not sharing a common superscript letter denote significant difference (*p <* 0.05).

The gut microbiota is an indispensable component for maintaining the normal physiological functions of the intestine. Gut microbiota dysbiosis is a pivotal factor in the pathogenesis and development of intestinal inflammation [28]. To investigate how curcumin and tryptophan modulate the gut microbial community, we performed 16S rRNA gene sequencing on cecal contents. Compared with the NC group, the Shannon, Simpson, Chao1 and Observed features index were significantly lower following DSS treatment. Consistent with the observed pathology, DSS treatment markedly reduced the α-diversity of the cecal microbiota. However, this loss of diversity was significantly counteracted by the therapeutic interventions (Figure 7A–D). Notably, the combined Cur–Trp supplementation was superior to either single-agent treatment, demonstrating the most potent effect in restoring a diverse and balanced microbial community.

The Venn diagram showed the distribution of Operational Taxonomic Units (OTUs) across the different groups (Figure 7E). A total of 2092 OTUs were identified across all five groups, of which 347 were shared among them. The number of unique OTUs specific to each group was 410 for the NC group, 291 for the Model group, 252 for the Cur group, 184 for the Trp group, and 309 for the Cur–Trp group. Next, β-diversity analysis was conducted to assess the overall structural similarity of the microbial communities. Non-metric multidimensional scaling (NMDS) analysis based on Bray–Curtis dissimilarity revealed a clear separation of the Model group from all other groups, which indicated a significant shift in its microbial composition (Figure 7F). Importantly, the Cur–Trp group clustered more closely to the NC group than either the Cur or Trp single-treatment groups. This suggested that the combination therapy was more effective at restoring the gut microbiota structure towards a healthy state.

At the phylum level, the gut microbiota was predominantly composed of *Bacteroidetes*, *Firmicutes*, *Proteobacteria*, *Verrucomicrobia* and *Proteobacteria* (Figure 7G). The uptake of DSS induced a significant shift in this composition, characterized by an increased relative abundance of Bacteroidetes and a concurrent decrease in Firmicutes. This led to a sharp decline in the *Firmicutes*/*Bacteroidetes* (F/B) ratio in the Model group compared to the NC group (Figure 7I). The *Firmicutes*/*Bacteroidetes* ratio has been proven to have a significant correlation with the state of the human gut microbiota [29]. All therapeutic interventions worked to normalize this ratio. The intervention of Cur–Trp (1.35 ± 0.13; Figure 7I) significantly improved the ratio (F/B) compared with the NC group (0.93 ± 0.11; Figure 7I).

At the genus level, the NC group was dominated by beneficial bacteria such as *Lachnospiraceae_NK4A136_group*, followed by *Bacteroides* and *Akkermansia* (Figure 7H). Compared to the healthy NC group, the abundance of *Lachnospiraceae_NK4A136_group* was significantly depleted in the DSS-induced Model group. All therapeutic interventions, however, worked to counteract this reduction. While both the Cur and Trp groups led to a partial recovery, Cur–Trp therapy demonstrated the most potent effect, restoring the abundance of this beneficial genus to a level closest to that of the NC group. The supplementation of Cur–Trp could reverse these changes. This suggests a possible direct link between the beneficial shifts in gut microbiota composition and the enhanced production of key microbial metabolites.

To identify specific microbial taxa that were differentially abundant among the groups, we performed Linear Discriminant Analysis Effect Size (LEfSe). This analysis identified 52 discriminative taxa from the phylum to the genus level (LDA score > 3.0) (Figure 7N,O). In the Model group, the analysis highlighted a significant enrichment of taxa belonging to the phylum Bacteroidota, including the class Bacteroidia and the order Bacteroidales. At the genus level, *Lachnospiraceae_UCG-006* was also significantly enriched in this group. The Cur–Trp group was characterized by the enrichment of 11 distinct taxa. Notably, the therapeutic impact on the microbiota was distinct across the treatment groups. While the Cur and Trp monotherapies respectively enriched specific beneficial genera, such as *Akkermansia* and *Azospirillum*, the Cur–Trp therapy uniquely fostered the growth of a different set of key commensals, including the butyrate producers *Oscillibacter* and *Intestinimonas*, as well as the mucus-associated *Mucispirillum schaedler.* To complement the NMDS analysis, Analysis of Similarities (ANOSIM) was conducted to statistically test for differences in microbial community structure. The results confirmed significant dissimilarities among the groups (R > 0, *p* < 0.01) (Figure 7P). The enrichment of these beneficial bacteria in the Cur–Trp group suggests that the combined intervention effectively reshapes the gut microbiota towards a healthier, SCFA-producing profile, contributing to the observed therapeutic outcomes. We further analyzed the complex interactions between gut microbiota, host physiological markers, and microbial metabolites using a Spearman heatmap (Figure 7Q). Specifically, the abundance of *Bacteroides* and *Escherichia-Shigella* as opportunistic pathogens was significantly positively correlated with key pathological markers of colitis such as MPO, MDA, TNF-α, IL-6, and IL-1β. Conversely, it was significantly negatively correlated with the level of SCFAS. In contrast, beneficial bacteria such as butyric acid-producing nuclei *Blautia* and *Oscillibacter*, and propionic acid-producing *Muribaculum* showed an opposite correlation pattern, which was significantly negatively correlated with the above colitis-related pathological markers (MPO, MDA, TNF-α, IL-6, IL-1β), and significantly positively correlated with SCFA levels.

## 4. Discussion

This study represents the first comprehensive investigation of the synergistic therapeutic effects of curcumin and tryptophan in a DSS-induced model of ulcerative colitis (UC) in mice. Our findings clearly indicate that the combined administration of these two compounds significantly improved the macroscopic symptoms of colitis, including prevention of weight loss, reduction of the disease activity index (DAI), and alleviation of colon shortening, while also effectively restoring intestinal barrier function and profoundly modulating the gut microbiota towards a healthier and more balanced state. These observations collectively highlight a multi-dimensional mechanism of action, which we will discuss in detail, emphasizing the intricate interactions among the host, its microbiota, and the therapeutic components.

### 4.1. Curcumin and Tryptophan Synergistically Mitigate Inflammation and Oxidative Stress

The synergistic anti-inflammatory effects of curcumin combined with tryptophan treatment are manifested at multiple molecular levels. Curcumin, a well-established anti-inflammatory agent, has been widely reported to exert its effects by influencing key inflammatory pathways, including the NF-κB signaling pathway [30]. According to these known characteristics, our research observed that curcumin alone significantly reduced the levels of pro-inflammatory cytokines such as TNF-α, IL-1β, and IL-6, a result consistent with its established role in alleviating excessive inflammatory signaling. Notably, when curcumin was administered in combination with tryptophan, this inhibitory effect on pro-inflammatory factors was greatly enhanced, suggesting that tryptophan may complement the action of curcumin by strengthening the suppression of pro-inflammatory pathways [31].

A significant finding of this study is the notable upregulation of anti-inflammatory cytokines, particularly IL-4 and IL-10, following co-administration of curcumin and tryptophan. The levels of these beneficial cytokines exceeded those observed when either compound was administered alone, suggesting that the two compounds work together to maintain inflammatory homeostasis through different but complementary mechanisms. This observation suggests that curcumin and tryptophan rebalance the inflammatory microenvironment in complementary ways—each component may help regulate distinct but interrelated molecular processes that work together to promote inflammatory homeostasis [32]. For example, previous studies have shown that tryptophan and its metabolites can support anti-inflammatory responses by modulating immune cell function and cytokine production, while curcumin has been shown to enhance anti-inflammatory factor expression through various regulatory mechanisms [33]. The elevation of IL-4 and IL-10 observed in our study is consistent with these reported features of these two components, supporting the idea that their combination amplifies anti-inflammatory effects through complementary pathways. In addition, the combination therapy significantly restored oxidative homeostasis, as evidenced by reduced levels of MDA and MPO and increased SOD activity. This result suggests that curcumin and tryptophan not only synergistically inhibit the inflammatory response, but also jointly reduce oxidative damage, reflecting a comprehensive protective effect against the dual insult of inflammation and oxidative stress in colitis.

### 4.2. Curcumin and Tryptophan Synergistically Restore Intestinal Barrier Function

Intestinal barrier dysfunction is a core pathological feature of UC, encompassing the disruption of tight junction proteins, the mucus layer, and the immune barrier [34]. A systematic evaluation of the restorative effects of the combined treatment on intestinal barrier function was conducted. FITC-dextran permeability assays and immunofluorescence detection of tight junction proteins were utilized to this end. The results of this evaluation were compelling. The combination treatment demonstrated a significant reduction in intestinal permeability and a marked increase in the expression of ZO-1, occludin, and MUC-2. This combination exhibited superior effects in comparison to either monotherapy. The mechanisms underlying this synergy are likely multifaceted. Firstly, curcumin has been demonstrated to protect tight junction proteins from oxidative damage by reducing the production of ROS [35]. Secondly, tryptophan metabolites directly promote the transcription of genes encoding tight junction proteins and mucins via the activation of the AhR signaling pathway [36]. Notably, the expression of MUC-2, the primary component of the mucus barrier, was significantly increased in the Cur–Trp group. MUC-2 not only provides a physical defense against pathogens but also supports microbial homeostasis by nourishing beneficial bacteria [37]. This indicates that the combined therapy creates a favorable environment for the reconstruction of the intestinal microbiota by strengthening the mucus barrier.

### 4.3. Curcumin and Tryptophan Synergistically Regulate Gut Microbiota Homeostasis

Gut microbiota dysbiosis is an important driving factor in the pathogenesis of UC, and therefore the main focus of treatment [38]. This study adopted 16S rRNA sequencing methodology to fully describe the regulatory effects imposed by combined curcumin and tryptophan treatments on gut microbiota structure as well as bringing out huge synergistic effects. With respect to α-diversity, the Shannon, Simpson, and Chao1 indices for the combination treatment group were significantly higher than those obtained for each monotherapy group separately, drawing nearer to values found within a normal control group [39]. β-diversity results assessed using NMDS indicated that microbiota structure from a combination treatment group was more similar to that from a normal control group.

This finding implied that combination therapy may have the potential to reverse DSS-induced microbiota dysbiosis more powerfully. Concerning the composition of microbiota, combination therapy significantly enhanced the *Firmicutes*/*Bacteroidetes* ratio, which is an index indicating intestinal health [40]. At the genus level, combination treatment significantly increased the abundance of beneficial bacteria such as *Lachnospiraceae_NK4A136_* group while reducing the relative abundance of potential pathogens such as certain Bacteroides species. The LEfSe test identified characteristic microbes in each group; most importantly, it indicated enrichment with butyrate-producing *Oscillibacter* and was beneficial because the produced butyrate not only served as a major fuel source for colonal epithelial cells but also had anti-inflammation and barrier effects [2], explaining why the short-chain fatty acid content in the combination therapy group was significantly higher than in other single therapy groups.

It is therefore reasonable to propose a “component–microbiota–host” interaction model. Specifically, curcumin is hypothesized to directly improve the intestinal microenvironment via its anti-inflammatory and antioxidant properties; this creates favorable conditions for the colonization and proliferation of beneficial bacteria capable of metabolizing tryptophan [41]. Based on existing research, under a healthy gut microbiota state, tryptophan tends to be converted into more indole-type metabolites in vivo. These endogenous metabolites, in turn, contribute to inflammation inhibition and intestinal barrier repair, effects that further support the maintenance of a balanced microbiota, forming a positive feedback loop [42]. Notably, the rapid anti-inflammatory action of curcumin provides a temporal window and favorable conditions for the gut microbiota to metabolize tryptophan. In contrast, the indole metabolites generated from tryptophan metabolism exert sustained effects, which help maintain long-term intestinal homeostasis following combination therapy. The rapid anti-inflammatory action of curcumin creates temporal space and opportunities for tryptophan metabolism by the microbiota, while continuous effects from indole metabolites would continue to maintain intestinal homeostasis in the long term after combination therapy. This temporal relationship is considered important for the enhanced therapeutic effect. Curcumin and tryptophan have a strong safety profile, largely due to their natural occurrence in common foods [43]. They prompt great strategies in the creation of new functional foods or dietary supplements. Challenges include dosage optimization on different formulation approaches toward increasing the already very low oral bioavailability of curcumin. The present study primarily employed an acute DSS-induced colitis model, which may not fully recapitulate the pathological features of chronic UC in humans.

### 4.4. Limitations and Future Directions

This study systematically investigated the synergistic therapeutic effects of curcumin and tryptophan on DSS-induced UC in mice. Key findings support the efficacy of this combination in alleviating UC and propose a ‘Component–Microbiota–Host’ interaction model that provides a theoretical framework. Given their natural dietary sources and safety advantages, both components also show potential as targeted functional foods for UC. However, this study has notable limitations that need to be addressed, along with corresponding future research directions. First, it relies on an acute DSS colitis model. Although this model helps study acute inflammation, it cannot fully simulate the chronicity and pathological complexity of human UC, limiting the generalizability of the results. Future research should validate the findings in chronic colitis models to assess long-term efficacy and safety. Secondly, although the results suggest the involvement of pathways such as AhR and NF-κB, no direct molecular evidence has been collected, such as the expression of downstream markers IL-22, CYP1A1, p65, and IκBα, which requires in-depth mechanistic studies to clarify. Third, although the composition of the microbiome has changed and the levels of short-chain fatty acids have increased, the direct causal relationship between specific microorganisms and these metabolites remains unconfirmed and requires further analysis. Fourth, the low oral bioavailability of curcumin may hinder its efficacy; it is essential to optimize dosages and develop formulation strategies to improve this. Finally, individual variations in the human gut microbiome may affect treatment outcomes, making it necessary to study personalized intervention strategies for clinical applications.

## 5. Conclusions

In this study, we demonstrated the potential of the combination of curcumin and tryptophan in intervening in DSS-induced ulcerative colitis (UC) in mice. Specifically, this combination alleviated the clinical symptoms of UC, restored intestinal barrier function, reduced oxidative stress and inflammatory responses, and significantly regulated the homeostasis of the gut microbiota, with effects more pronounced than the use of either component alone. This research provides direct experimental evidence for the effectiveness of the curcumin–tryptophan combination in intervening mouse UC, supporting further exploration of its applications in UC intervention. Furthermore, our findings offer new insights for developing dietary ingredient-based UC nutritional combination therapies, which may aid in the application of precise nutritional strategies in the management of inflammatory bowel disease (IBD).

## Figures and Tables

**Table 1:** Scoring system for disease activity index (DAI) in DSS-induced colitis in mice.

Score	Loss of Body Weight (%)	Loose Stools	Bloody Stools
0	<1	Olid and granular	Negative expression of occult blood
1	1–5	Soft and granular	Weakly positive expression of occult blood
2	5–10	Semi-formed loose stool	Positive expression of occult blood
3	10–15	Unformed loose stool	Strongly positive expression of occult blood
4	≥15	Had sign of liquid	Strongly positive and visible blood

**Table 2:** Histological scoring criteria for colonic injury.

Inflammation	Depth of Lesion	Cryptic Destruction	Extent of Disease (%)	Score
-	-	-	-	0
Mild	Mucosal layer	1/2	1–25	1
Moderate	Submucosa	2/3	26–50	2
Severe	Muscular and serosal layers	100%	51–75	3
-	-	Destruction of all crypts and intestinal epithelium	76–100	4

## Data Availability

The original contributions presented in this study are included in the article/Supplementary Material. Further inquiries can be directed to the corresponding author.

## Supplementary Materials

The following supporting information can be downloaded at: https://www.mdpi.com/article/10.3390/nu17182988/s1, Figure S1. Standard curve of SCFA. (A) Acetic acid; (B) Butyrate acid; (C) Propionic acid; (D) Isovaleric acid; (E) Isobutyric acid; (F) valeric acid.
