# Supplementary material for: Curcumin Combined with Tryptophan Ameliorates DSS-Induced Ulcerative Colitis via Reducing Inflammation and Oxidative Stress and Regulation of Gut Microbiota
Source: Nutrients. 2025 Sep 18;17(18):2988. doi: 10.3390/nu17182988 (PMC12472673; doi:10.3390/nu17182988)
Supplement: Supplementary file 1 [file nutrients-17-02988-s001.zip › nutrients-3862288-supplementary.pdf]

Supplementary Materials

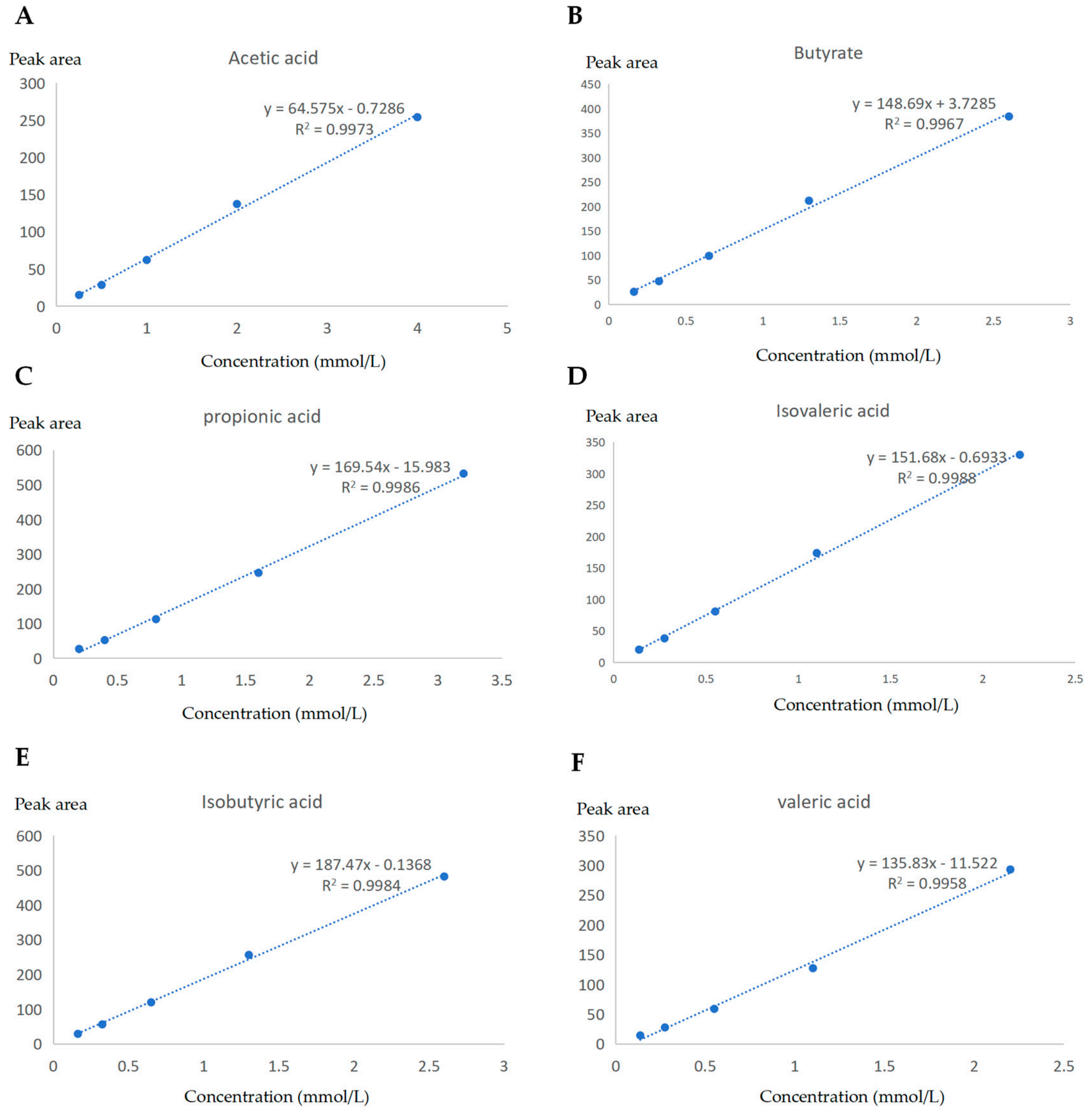

Figure S1. Standard curve of SCFA. (A) Acetic acid; (B) Butyrate acid; (C) Propionic acid; (D) Isovaleric acid; (E) Isobutyric acid; (F) valeric acid.
